# Building implementable packages for universal health coverage

**DOI:** 10.1136/bmjgh-2022-010807

**Published:** 2023-05-17

**Authors:** Teri Reynolds, Thomas Wilkinson, Melanie Y Bertram, Matthew Jowett, Rob Baltussen, Awad Mataria, Ferozuddin Feroz, Mohamed Jama

**Affiliations:** 1 Department of Integrated Health Services, World Health Organization, Geneva, Switzerland; 2 World Bank Group, Washington, District of Columbia, USA; 3 Health Systems Governance and Financing, World Health Organization, Geneva, Switzerland; 4 Health Financing and Governance, World Health Organization, Geneva, Switzerland; 5 Department for Health Evidence, Radboudumc, Nijmegen, The Netherlands; 6 Department of Universal Health Coverage/Health Systems, World Health Organisation Regional Office for the Eastern Mediterranean, Cairo, Egypt; 7 Islamic Republic of Afghanistan Ministry of Public Health, Kabul, Afghanistan; 8 Federal Government of Somalia, Mogadishu, Somalia

**Keywords:** Public Health, Health economics, Health services research, Health systems

## Abstract

Since no country or health system can provide every possible health service to everyone who might benefit, the prioritisation of a defined subset of services for universal availability is intrinsic to universal health coverage (UHC). Creating a package of priority services for UHC, however, does not in itself benefit a population—packages have impact only through implementation. There are inherent tensions between the way services are formulated to facilitate criteria-driven prioritisation and the formulations that facilitate implementation, and service delivery considerations are rarely well incorporated into package development. Countries face substantial challenges bridging from a list of services in a package to the elements needed to get services to people. The failure to incorporate delivery considerations already at the prioritisation and design stage can result in packages that undermine the goals that countries have for service delivery. Based on a range of country experiences, we discuss specific choices about package structure and content and summarise some ideas on how to build more implementable packages of services for UHC, arguing that well-designed packages can support countries to bridge effectively from intent to implementation.

**Summary box**

- Essential packages of health services (EPHS) only have impact when implemented, yet in many countries there is still a large implementation gap. Critical translational work is needed to move from criteria-driven formulations to packages designed to support implementation.
- This study identifies key strategic choices that countries can make in package design to facilitate successful delivery of the health services included in an EPHS. Package design elements that support service delivery include use of a rationalised architecture of interventions with consistent granularity, entries expressed as services rather than diseases, specification of local delivery platforms and assignment of services to platforms and visualising linkage to burden of disease.
- This article demonstrates how service delivery considerations can be integrated into package development and how package documents can be structured and formulated to make them context specific and implementable. WHO has a range of tools to support these country efforts.

## Background

The central tenet of universal health coverage (UHC) is that all people should have the high-quality care they need without suffering financial hardship.^1^ Since no country or health system can provide every possible health service to everyone who might benefit, the idea of prioritising a defined subset of services for universal availability is intrinsic to UHC. Strongly, though not inevitably, linked to the UHC agenda is the idea that this prioritisation process should be executed according to certain principles. The WHO recommends that prioritisation be based on explicit criteria and that it should incorporate consideration of service delivery realities.^2^ Given limited individual resources in most contexts, the UHC requirement for financial protection usually carries with it an implication that public funds will be preferentially directed towards these prioritised services (see Soucat *et al* in this series),^3^ and in some contexts, the explicit goal is to make these services free at the point of care. In general, population access to services intended for UHC may be protected through a variety of government assurance mechanisms, including but not limited to direct financing or direct provision for some groups, mandatory contribution and prepayment schemes and regulatory structures that constrain what public and private entities pay for or deliver. There are, of course, many other kinds of health service packages and subpackages, designed for a range of uses. Here, we focus exclusively on packages of priority services intended for UHC (in this series, referred to as an essential package of health services (EPHS)). The component services of an EPHS are sometimes also referred to more generically as interventions, and they span a wide scope of healthcare activities that depend in turn on specific health system inputs, such as health workforce, medications, devices, protocols and other resources.

The elaboration of an EPHS, however, even when done in perfect accordance with the principles above, does not in itself benefit a population—packages have impact only through implementation. Despite increasing country interest in (and improving processes for) EPHS development and revision in recent years, a vast implementation gap remains, with coverage of many essential services remaining low even when they are included in an EPHS.^4–6^

Failures of implementation are often attributed to financial resource limitations, but not all barriers are economic. There are inherent tensions between the way services are formulated to facilitate criteria-driven prioritisation and the formulations that facilitate implementation, and service delivery considerations are rarely well incorporated into package development. As described below, when burden of disease is used as a criterion for inclusion in an EPHS, high-burden health conditions, for example, must be ‘translated’ or mapped to the services that address them; and interventions taken from a series of independent cost-effectiveness studies (which may span from single drugs or procedures to entire programmes), for example, may be too heterogeneous to support a consistent delivery approach.

Countries face substantial challenges bridging from an EPHS list to the capacity-building, human and material resources, and organisational and financing elements needed to get services to people. They struggle to account for the interdependence of services and platforms, and to bridge from disease- or population-specific services to integrated service delivery. Finally, they struggle to create a coherent approach to people’s health needs from lists of individual interventions that often leave out the foundational demand-driven health services for common conditions that make up much of primary care.

The premise of this paper is that EPHSs are powerful mechanisms for influencing service delivery (for better or worse) and that there are key strategic choices that countries can make in package design and implementation approaches to facilitate successful delivery of the health services included in an EPHS. Well-designed packages can support countries to bridge effectively from prioritisation to implementation; at the same time, the failure to incorporate delivery considerations at the prioritisation and design stage can result in packages that undermine the goals that countries have for service delivery, particularly regarding integration and people centredness. Of course, well-designed packages are not sufficient in themselves to ensure successful implementation, but they are a necessary foundation and can help ensure that countries’ package development processes support their goals for service delivery.

Technical support for package implementation is increasingly identified as a high priority for countries, and here we aim to capture ideas on package design based on the authors’ experiences across many countries, including but not limited to those addressed by other papers in the series.^3 7–11^ Recognising that package implementation is highly context dependent, we focus on how considerations about service delivery can be incorporated into package structure and content to design more implementable packages.

## The uses and abuses of packages for UHC

### Progressive realisation, now or later

Even in highly constrained environments, a complex range of services is provided to different population groups. An EPHS does not seek to encompass all possible services offered by a given health system but explicitly outlines a subset of interventions to be offered universally at a defined level of quality based on need. An EPHS may be quite small to begin with, containing a limited set of interventions, and its contents progressively expanded over time, or an EPHS may be large from the beginning, containing a broad range of services beyond those widely available, and its implementation progressive. In the former case, the package can be operationalised in the near term; in the latter, it sets a horizon of policy intent as resources and capacities expand over time.

Packages that start small have a different relationship to implementation mechanisms than packages that start large (including services that stretch or exceed the current capacity of the system for service delivery). A smaller EPHS is more immediately ‘operational’ and is usually intended to be provided universally in the near term (and in this case, the health system as a whole may deliver much of its care outside the EPHS, such as through private sector services paid for by users). An expansive EPHS containing services that exceed current delivery capacity, on the other hand, aims to set goals for what the health system should achieve over time. Fundamentally EPHSs serve to address a mismatch between people’s health needs and the services available to them, but they do so in different ways in different policy contexts, and many countries’ packages are a blend of the operational and the aspirational.

Based on country experiences, whatever the initial intent for package development (and this varies among stakeholders), defined packages of services are ultimately used for many purposes.^12–18^ These include the more traditional use for defining entitlements, but packages are also used to define contracting responsibilities for service providers, and for budgeting purposes. They are widely used to support service planning, including as a foundation for health workforce competencies and training, and for material resource (and supply chain) planning. Finally, they may be used for programme reporting to communicate to donors and partners what is being done for particular diseases or subpopulations. Taken together, these uses confer the power of an EPHS to transform healthcare delivery, and each of these use cases has implications for the way packages should be designed to best meet country needs.

The most basic presentation of a package is a simple static list of services, perhaps organised by platform of care, health area or life course stage. While the exercise of collating these lists should be based on available evidence and accountable deliberative processes, the failure to consider major mechanisms of implementation while constructing the package at best limits the power of the package to drive improvements in health outcomes, and at worst undermines the values and goals that countries have for service delivery.

Delivering against an EPHS means bridging the gap from development to implementation, and this delivery process starts with the package itself. There is critical translational work to be done between traditional steps of EPHS development and successful implementation. To this end, approaches to service prioritisation, and the package documents that result, should not be primarily driven by what makes prioritisation most convenient, or by a theoretical construct of what ‘ought’ to inform relative trade-offs between competing investments, but by the goal of getting care to people and people to care. EPHSs should already incorporate the terminology, content and structure that will best support implementation. Otherwise, we risk uncoupling robust package development processes from the service delivery that gives them meaning.

## Building implementable packages

The purpose of an EPHS is to influence the care and services that people receive (in this sense, a package is also a plan), and this has substantial implications for how a package should be formulated. The process of implementation-oriented EPHS design is a deliberate adjustment from a historical pattern of service delivery to a model based on available evidence, deliberative processes and strategic policy direction.

The criteria commonly used for prioritisation, particularly burden of disease and cost-effectiveness, strongly shape package terminology and granularity and create interdependence among services. Criteria should be considered together and alone—equity may lead in a different direction than cost-effectiveness, cost-effectiveness formulations may not align with integrated service delivery and political priorities may be in tension with protection of the vulnerable or addressing the local burden of disease. Moreover, each of these criteria leads to interventions that are formulated in different ways. Movement from criteria-based formulations to implementation, therefore, has many pitfalls and must be actively managed. For example, identifying which services address what part of the burden of disease requires substantial interpretation, and often results in services formulated as diseases (‘management of asthma’ or simply ‘asthma’) that lack the specificity to be implemented (or costed for that matter). A service described as ‘initial evaluation and referral for X’ (a formulation that appears in several sources) may be effective and cost-effective only when the complementary referral service is available, but such complementary services are rarely considered as a linked prioritisation choice.

We believe that the reality of service delivery, including information about where services will be delivered and the interdependence among local service delivery platforms, must be considered and the terminology that derives from different selection criteria rationalised to create an implementable package—formulations from burden of disease lists, for example, are not the same as those from cost-effectiveness analysis (CEA) studies, and neither is optimal for successful implementation (see examples below). The structure of packages should support decision-making that incorporates relational health system aspects (eg, referral across platforms), and the content of packages should include foundational services and be expressed in terminology that reduces ambiguity and supports implementation.

### Package structure and content

Package terminology and structure affect the ability to implement and cost an EPHS. Formulations that lack detail, for example, can be interpreted in many ways and correspond to different resource requirements. For a given reference source to be applicable, the service implemented should be broadly the same as the service studied. Based on the experiences of the authors in many countries, there is rarely any mechanism to ensure that the intervention studied in a reference source is the one included in an implementation plan (‘management of ectopic pregnancy’, for example, might have been studied and deemed cost-effective as a bundle including medical and surgical management, when only surgical management is available in a given context). Indeed, interventions are often modified or adapted, and rarely are original evidence sources available or accessed during consultations, nor would this always be practical. If this limitation is not made explicit and managed, it can uncouple selection criteria and service delivery in ways that fundamentally affect the legitimacy of the package development and delivery processes.

Often because they aggregate formulations from heterogenous scholarly literature, standard references formulate lists of services that include single drugs or procedures, microprogrammes, diseases and subpopulations.^19^ This creates many challenges for implementation. It is difficult to articulate a stepwise implementation strategy that will work for both a single therapeutic (eg, ‘provision of cotrimoxazole to children born to HIV-positive mothers’) and a complex bundle comprised of many services (‘adolescent-friendly health services including: provision of condoms to prevent STIs; provision of reversible contraception; treatment of injury in general and abuse in particular; and screening and treatment for STIs’).^20^ It would be even more challenging to develop distinct implementation strategies for each of a list of highly variable package entries.

**Figure 1:**
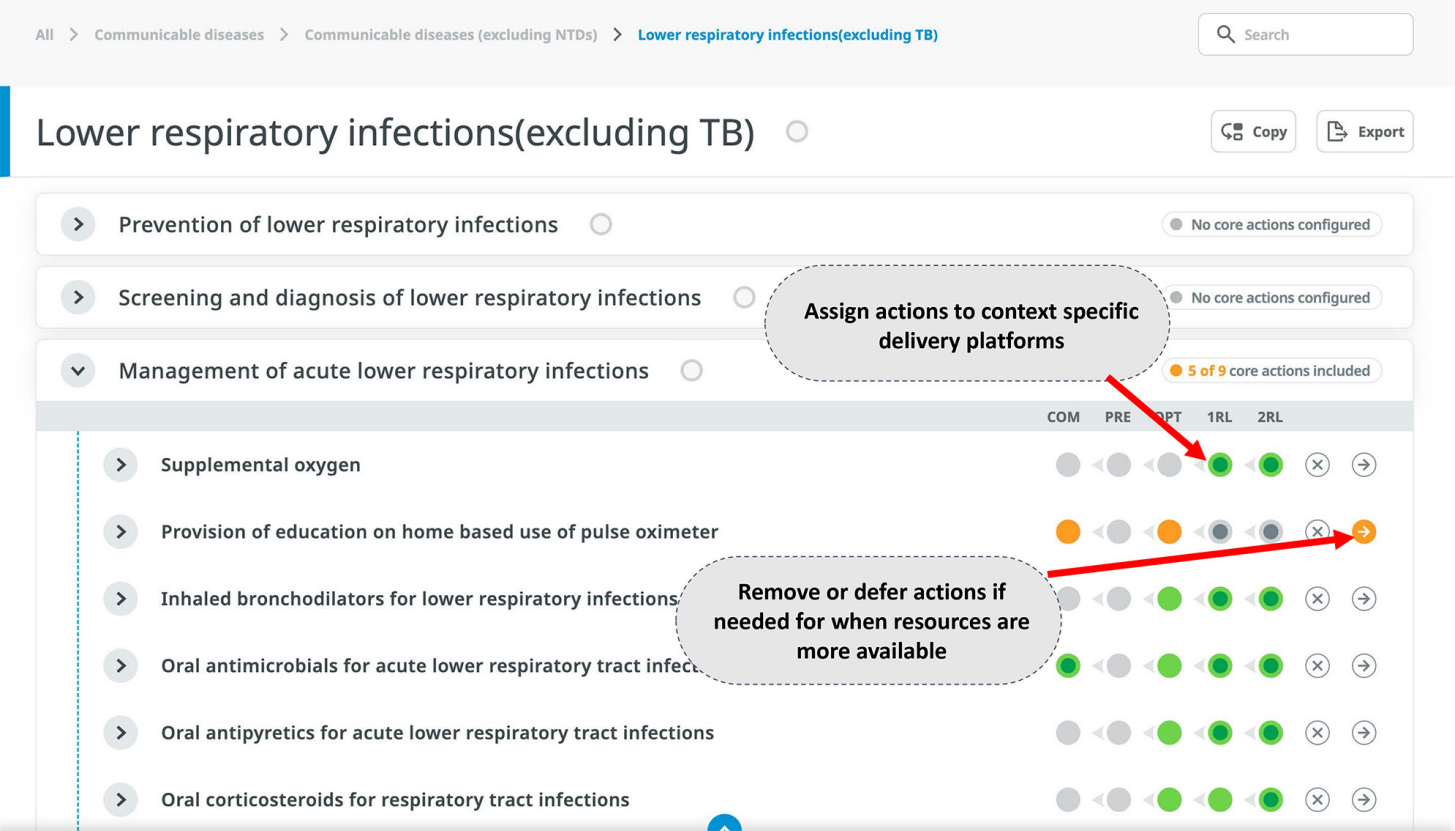
Example of services and platforms from the WHO Universal Health Coverage Service Package Delivery and Implementation (SPDI) tool (access at UHCC.who.int). TB, Tuberculosis.

**Figure 2:**
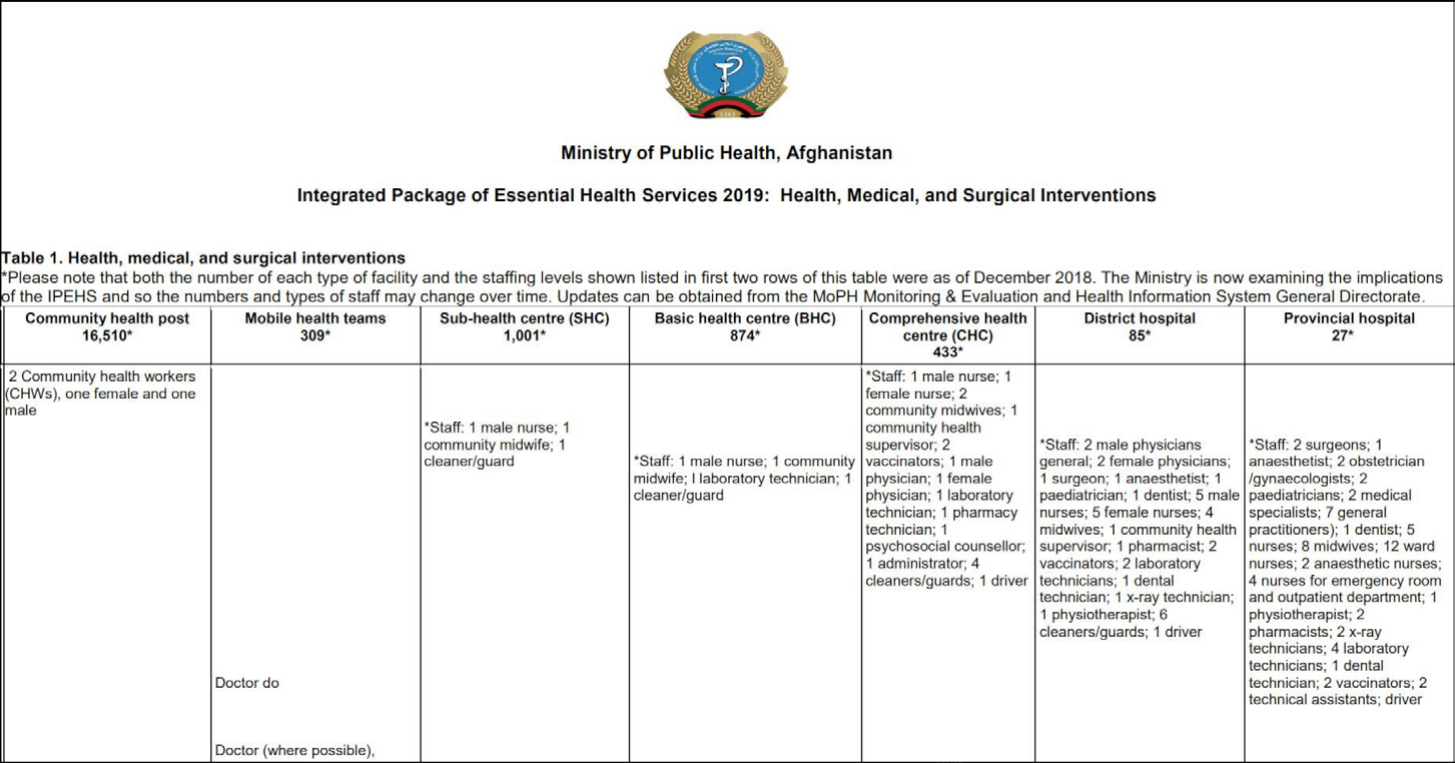
Example from the Islamic Republic of Afghanistan Ministry of Public Health (MoPH) Integrated Package of Essential Health Services (IPEHS) 2019.

**Figure 3:**
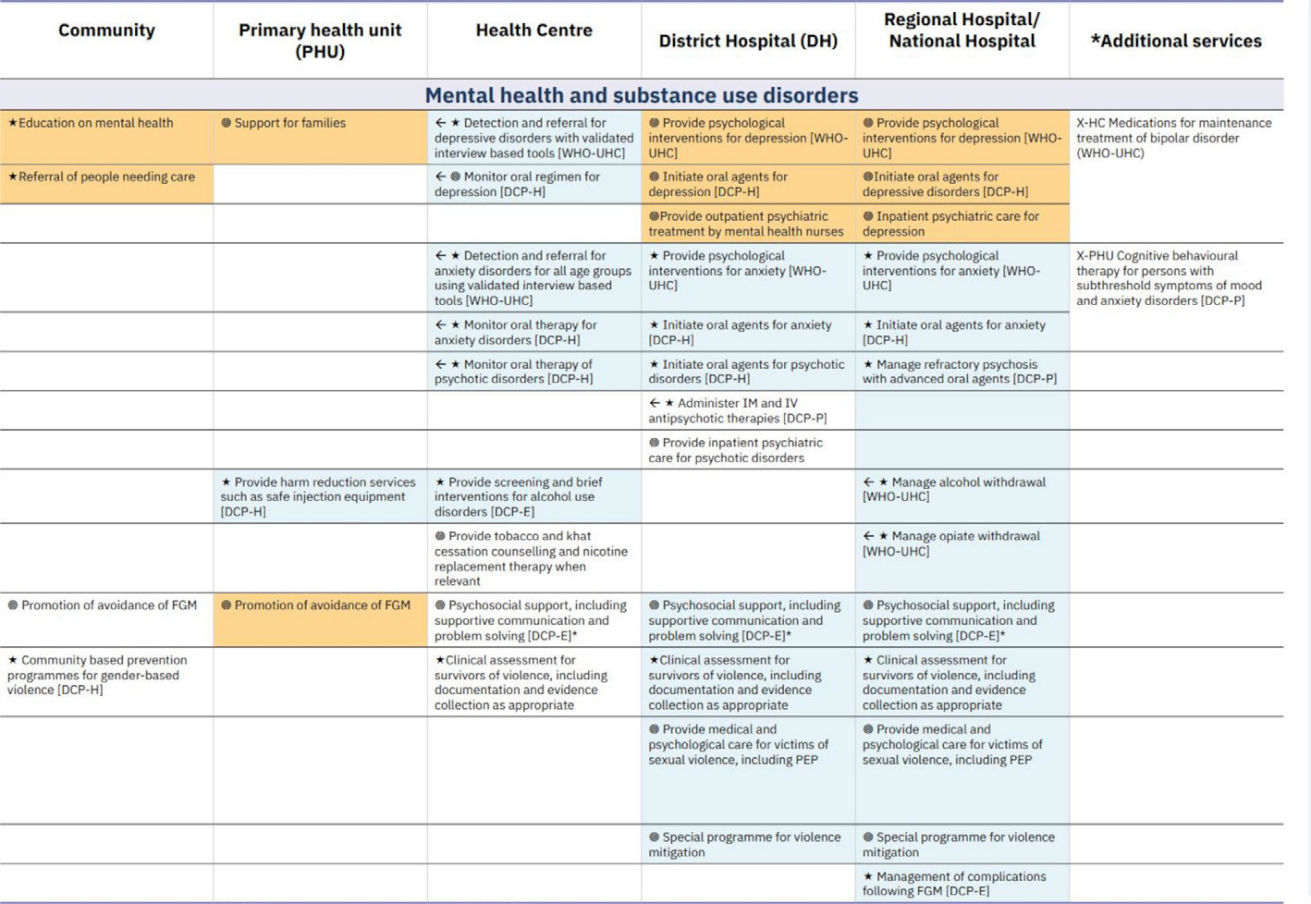
Example from the essential package of health services, Somalia, 2020. *Additional services are the interventions that, added to the services in the core package, constitute the extended package, to be progresively implemented when more resources become available. UHC, universal health coverage; NTD, Neglected tropical diseases; HBD is high-burden disease; DCP-H, Disease Control Priorities highest priority package.

**Figure 4:**
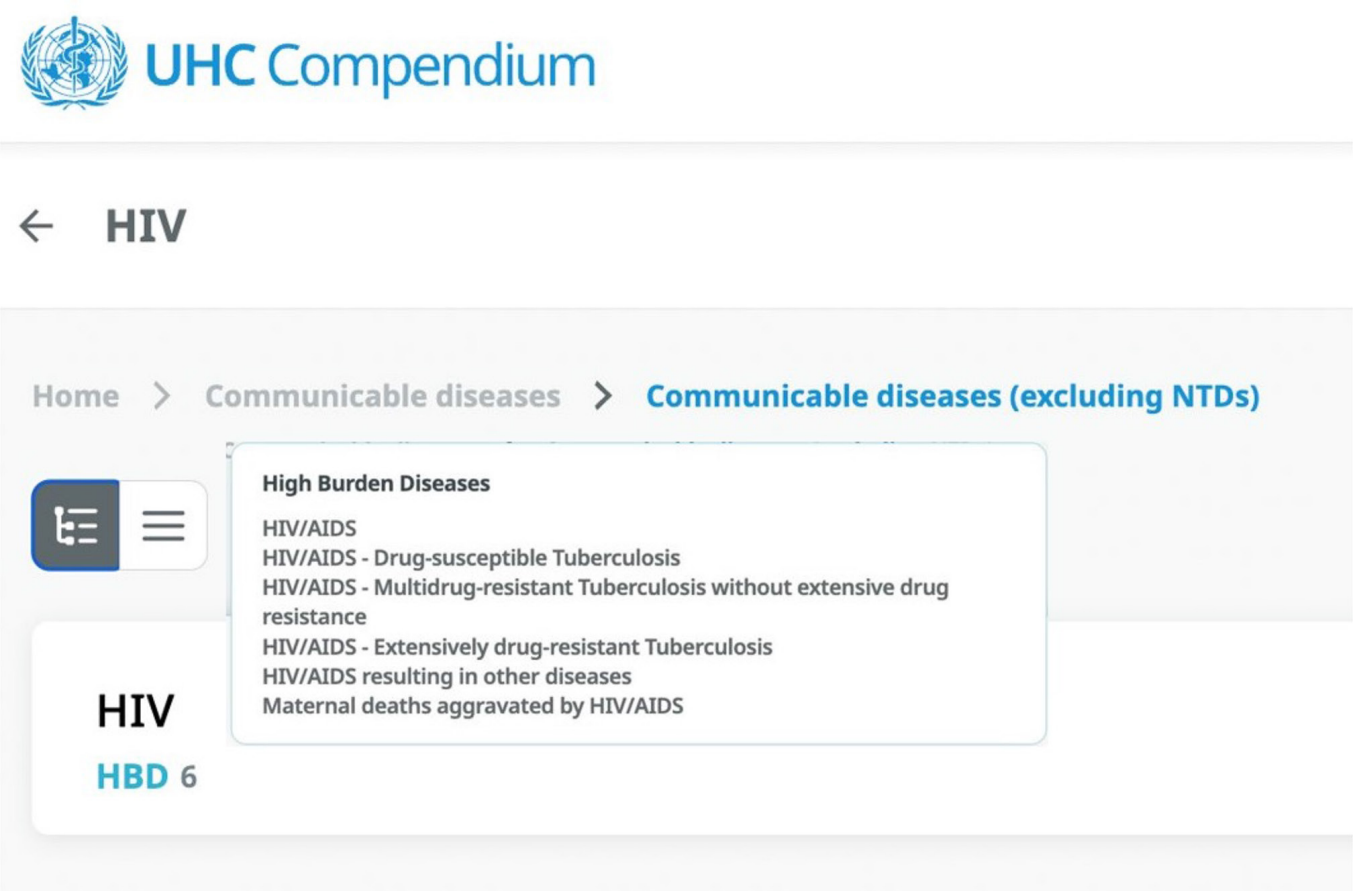
Example of services for a high-burden disease from the WHO Universal Health Coverage Service Package Delivery and Implementation (SPDI) tool (access at UHCC.who.int). NTD, Neglected tropical diseases; HBD, high-burden disease.

Package design elements that support implementation considerations during review, and can be included in a simple spreadsheet, include:

- Use of a rationalised architecture of interventions with consistent granularity and nested levels of granularity for different needs. This allows choices at the relevant level for different stages of prioritisation and assessment, allowing working groups to view or hide detail as needed) (see figure 1).
- Entries expressed as services rather than diseases. This supports translation to the delivery context, including assignment to service delivery platforms, monitoring, mapping of health worker competencies and other uses such as coverage estimation). Examples include ‘external haemorrhage control with tourniquet’ or ‘internal fracture fixation’ rather than ‘treatment of injury’ in general.
- Specification of local delivery platforms and assignment of services to platforms. Adding local information on staffing norms, for example, can guide decisions and foreground feasibility considerations to ensure that the total list of services assigned to a given platform is appropriate. See figures 1–3 for examples from WHO, Afghanistan and Somalia.
- Use of a structure that visually represents relationships among platforms (eg, by aligning related interventions across rows) to ensure that interdependent interventions (eg, lower-level services that depend on higher level services for their effectiveness and cost-effectiveness) are always seen, reviewed and prioritised together. See figures 1–3 for examples from WHO, Afghanistan and Somalia.
- Visualising linkage to burden of disease, such as colour coding for services addressing top causes of death and disability. This allows simultaneous consideration of this criterion while others are discussed and supports prioritisation of services and designation of delivery platforms that match the country’s health needs. See figures 3 and 4 for examples from Somalia and WHO.
- Using symbols that indicate a progressive horizon for implementation, such as arrows to indicate a shift from the initial platform where a new service might be introduced to the optimal platform for delivery once capacity or funding is available. See figures 1 and 3 for examples from Somalia and WHO.
- Using formulations that have adequate detail and are organised to support mapping to the human and material resources required for implementation (see figure 4).

### CEA and integration: the whole and the sum of its parts

There is increasing country interest in including interventions that are proven to be effective and cost-effective,^21–23^ but there is some mismatch between the techniques and goals of CEA for a specific intervention in a defined context and the techniques and goals of defining a package of priority services for UHC. Challenges with the development and use of CEA data have been described by Baltussen *et al* in this series,^8^ but here we focus on some of the challenges their use raises for implementation.

CEA evidence can be effectively used to identify marginal choices that provide the most benefit within a limited resource envelope, but there are important limitations. CEA studies are often oriented to support decisions about incremental additions to an existing system and are necessarily highly contingent on context. While CEA evidence transferred to another setting can provide directional indication of the likely relative cost effectiveness of interventions, CEA cannot provide an absolute quantification of likely costs and benefits across contexts. CEA data alone cannot support a framework for allocatively efficient distribution of resources.

**Figure 5:**
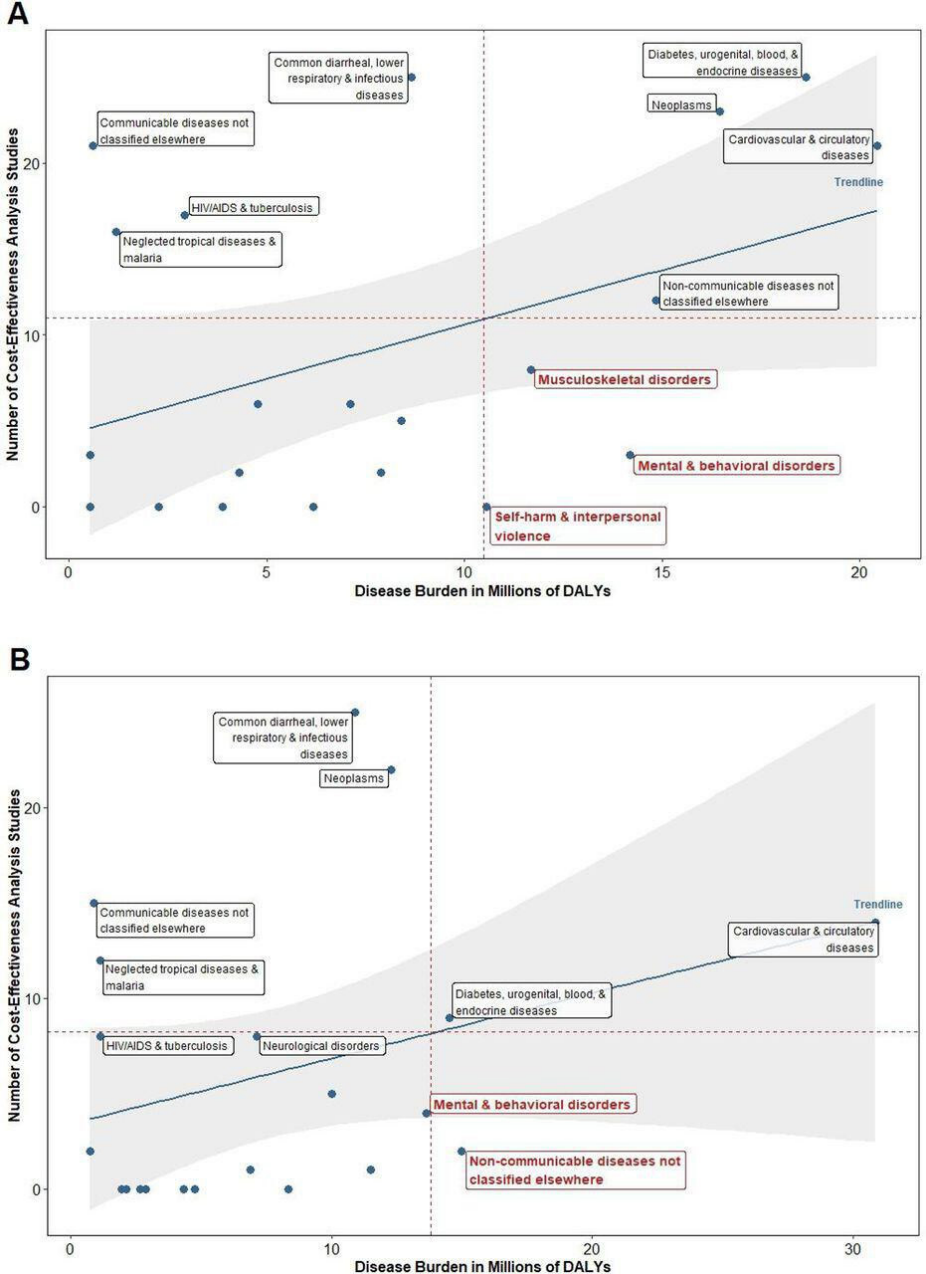
Number of cost-effectiveness analyses versus disease burden for selected diseases: (A) Latin American and the Caribbean and (B) North Africa and the Middle East. Adapted from Do *et al*.^29^ DALY, Disability-adjusted life years.

In addition, CEA literature does not cover the full possible list of services that should be considered for an EPHS. Indeed, CEA’s strategic focus of ‘incremental additions’ often prioritises areas of uncertainly or controversy and deprioritises analyses of foundational services that make up the bulk of promotive and demand-driven primary care. A large proportion of facility visits are never linked to a specific diagnosis, but are based on symptoms that are assessed, managed and often resolved without a diagnosis ever being made.^24–27^ These services are (or are often considered to be) so widely available in well-resourced settings that they do not require a ‘decision’ on inclusion, and they are rarely represented in CEA studies. Even for specific conditions, there is a mismatch between burden of disease and volume of CEA studies published (see figure 5 below). Even among the 218 interventions included in the Disease Control Priorities 3 Essential UHC package, 89 lack supporting CEA evidence.^28 29^

Bringing together a range of incremental services, particularly when skewed toward newer and emerging services, neither creates a coherent whole nor provides a solid foundation for package development in limited-resource settings. Many services will need to be part of an EPHS even if they have not been—or cannot be—quantified through a cost-effectiveness lens. Consideration for integrated service delivery and its benefits through economies of scope and scale is also an important criterion. To build coherent and implementable packages for UHC, we must already think towards their ultimate use and complement CEA considerations by incorporating service delivery considerations into the structure and content of the packages themselves.

## Conclusion

Packages of priority services are intrinsic to the idea of UHC and are powerful mechanisms for influencing service delivery. Despite increasing attention to service package development, however, countries continue to struggle with implementation. Delivering a package means bridging the gap from development to implementation, and this delivery process starts with the package itself. Critical translational work is needed to move from criteria-driven formulations to packages designed to support implementation.

Effective package design will not, of course, ensure effective implementation—designing an implementable package is a necessary but not sufficient passage on the road to UHC. There are key strategic choices that countries can make in package design to facilitate successful delivery, and this paper aggregates some key ideas from country experiences. Based on these experiences, WHO has developed the UHC Compendium of health interventions and the associated Service Package Delivery and Implementation Tool (see www.UHCC.who.int), which incorporates the content and structural elements described in this paper to support countries to take a structured approach to building implementable packages.^30^ Approaches to service prioritisation, and the package documents that result, should orient to impact and incorporate the terminology, content and structure that will best support implementation, creating packages aligned with the way countries use them and that improve the care people receive.

## Data Availability

No data are available.
